# Awake ^18^F-FDG PET Imaging of Memantine-Induced Brain Activation and Test–Retest in Freely Running Mice

**DOI:** 10.2967/jnumed.118.218669

**Published:** 2019-06

**Authors:** Alan Miranda, Dorien Glorie, Daniele Bertoglio, Jochen Vleugels, Guido De Bruyne, Sigrid Stroobants, Steven Staelens, Jeroen Verhaeghe

**Affiliations:** 1 Molecular Imaging Center Antwerp, University of Antwerp, Antwerp, Belgium; 2 Product Development, University of Antwerp, Antwerp, Belgium; and; 3 University Hospital Antwerp, Antwerp, Belgium

**Keywords:** neurology, PET, animal behavior, motion correction, mouse brain, positron emission tomography

## Abstract

PET scans of the mouse brain are usually performed with anesthesia to immobilize the animal. However, it is desirable to avoid the confounding factor of anesthesia in mouse-brain response. **Methods:** We developed and validated brain PET imaging of awake, freely moving mice. Head-motion tracking was performed using radioactive point-source markers, and we used the tracking information for PET-image motion correction. Regional ^18^F-FDG brain uptake in a test, retest, and memantine-challenge study was measured in awake (*n* = 8) and anesthetized (*n* = 8) C57BL/6 mice. An awake uptake period was considered for the anesthesia scans. **Results:** Awake (motion-corrected) PET images showed an ^18^F-FDG uptake pattern comparable to the pattern of anesthetized mice. The test–retest variability (represented by the intraclass correlation coefficient) of the regional SUV quantification in the awake animals (0.424–0.555) was marginally lower than that in the anesthetized animals (intraclass correlation coefficient, 0.491–0.629) over the different regions. The increased memantine-induced ^18^F-FDG uptake was more pronounced in awake (+63.6%) than in anesthetized (+24.2%) animals. Additional behavioral information, acquired for awake animals, showed increased motor activity on a memantine challenge (total distance traveled, 18.2 ± 5.28 m) compared with test–retest (6.49 ± 2.21 m). **Conclusion:** The present method enables brain PET imaging on awake mice, thereby avoiding the confounding effects of anesthesia on the PET reading. It allows the simultaneous measurement of behavioral information during PET acquisitions. The method does not require any additional hardware, and it can be deployed in typical high-throughput scan protocols.

Small-animal PET is performed under anesthesia to ensure immobilization of the animal and to avoid motion artifacts in the images. However, studies have indicated that anesthetics can interfere with the uptake of several PET radiotracers through the alteration of such physiologic parameters as cerebral blood flow, body temperature, and heart rate (1–3). Physical restraint of the awake animal during the PET acquisition has been proposed to circumvent these issues (4). Unfortunately, immobilization stress also affects the uptake of such radiotracers as ^18^F-FDG (5) and ^11^C-raclopride (6). Given these confounding factors, it is desirable to perform PET acquisitions on freely moving animals, to ensure an unaffected brain response.

Several methods have been proposed for obtaining PET scans of the brain of freely moving animals. Schulz et al. (7) surgically fixed a miniaturized PET scanner to the rat skull. An approach by Kyme et al. (8) used a commercially available PET scanner to perform PET acquisitions with minimal restraint. In the latter method, an optical device tracked the motion of the rat head during the acquisition, and motion correction was subsequently applied to the images. Our group developed this method further, replacing optical motion tracking with point-source tracking (PST) in which radioactive markers are fixed to the head of the rat to measure its movement (9). The PST method does not require any hardware other than the PET scanner, and it allows tracking to be performed throughout the entire field of view (FOV), regardless of animal pose or scanner-bore size.

Earlier studies have been based only on PET imaging performed on awake rats, although transgenic models commonly used in neuroscience are more widely available for mice (10). For this reason, we report on the adaptation of the PST method to track head motion in freely moving mice during PET acquisitions. Compared with rats, the space available on the mouse head to fix the point sources is limited. In addition, motion range and speed are greater in mice than rats. These aspects will be considered when adapting the tracking algorithm for mouse imaging.

In this study, we compared the imaging of awake mice using PST versus the imaging of anesthetized mice. To this end, ^18^F-FDG was used to perform PET scans on a group of anesthetized mice that had undergone an awake tracer uptake period, as well as on a group of mice scanned in an awake state. Test–retest and memantine-induced brain activation studies (11) were performed on both groups. Given the irreversible uptake of ^18^F-FDG, we expected to observe similar brain uptake in both groups, because both groups had been allowed similar awake tracer uptake periods. The memantine challenge was included because the induced increased locomotion (12) represents a challenge for the motion-tracking algorithm. In addition, it allowed us to assess the ability of the awake-mouse imaging to detect memantine-induced alterations in brain uptake and in behavior.

## MATERIALS AND METHODS

### PET Scanner

Scans were performed using 2 Inveon scanners (Siemens Medical Solutions, Inc.). The axial FOV is 12.7 cm in length, with a transaxial diameter of 10 cm. The scanner has a spatial resolution of 1.5 mm at the center of the FOV, which increases in the radial direction to 3.5 mm at the edge of the FOV (13) because of the parallax effect. Images were reconstructed in a 128 × 128 × 159 matrix, with a voxel size of 0.776 × 0.776 × 0.796 mm in the *x, y,* and *z* directions, respectively.

### Mouse Holder

**FIGURE 1.**
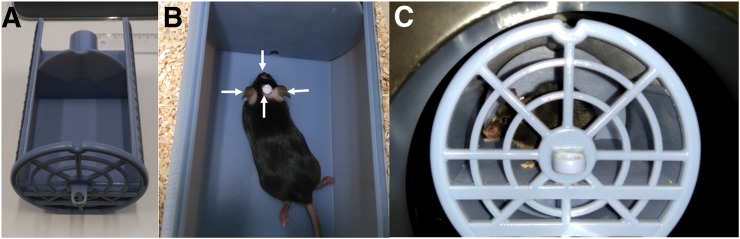
(A) Holder (top cover removed) in which mouse was placed during acquisitions. (B) Mouse with fixed point sources, indicated by arrows. (C) Mouse inside holder during PET acquisition.

A cylindric animal holder was designed to fit inside the Inveon scanner while keeping the mouse inside the scanner FOV (Fig. 1). The cylinder case has a length of 16.5 cm and a diameter of 10 cm. Inside the cylinder, a horizontal platform of 10 × 9 cm allows movement of the mouse in all directions.

### PST

We previously validated the PST method (9) for tracking the head motion of an awake rat. Briefly, 4 radioactive point sources (∼1 mm in diameter) are glued onto the animal’s head. Two point sources are fixed under each ear, one on the nasal bridge and one on top of the head on a lightweight spacer made of foam paper. The activity of each point source was in the range of 296–370 kBq. After the PET acquisition, the point sources are tracked in short-time-frame images (32 ms). Each short frame undergoes list-mode reconstruction (14). Each image frame is processed sequentially to determine the position of the point sources. Probable point sources are calculated, after which the correct point sources are found by selecting those that minimize a similarity error score with respect to a predefined model. Once the point sources have been found in all short frames, the pose (position and orientation) of the animal head is calculated from the point source locations. Poses are verified by aligning the frame point sources to the rigid model. Frames with at least one point source with a distance larger than 2 mm from the rigid model are discarded. Finally, motion-corrected PET images can be reconstructed. Compared with the rat head PST, the step at which the similarity to ideal spheres was calculated for point sources (9) had to be replaced. Because of the higher mouse motion speed, the point sources were blurred and did not represent ideal spheres. Therefore, a new metric that considers only geometric features, such as the position of the points with respect to other points, was included.

### Image Reconstruction

A list-mode ordered-subsets reconstruction (14) (16 subsets, 8 iterations) with spatial resolution modeling (15) was used for both motion-free (anesthetized mouse) and motion-corrected (awake mouse) images. Awake-mouse images included event-by-event motion correction (16). Attenuation correction factors were calculated from the CT scan for the anesthetized animals and from the mouse body activity outline for the awake animals (17). In the latter method, the mouse body shape is estimated from the whole-body uptake, which is present along the entire body for ^18^F-FDG. Attenuation by the mouse body is therefore considered in motion-corrected scans assuming a constant linear attenuation coefficient for soft tissue (0.097 cm^−1^) for the whole body. No scatter correction was performed. Dynamic reconstructions were performed as reconstructions of independent 2-min frames, using the same algorithms as for static reconstructions.

### Animal Preparation

**FIGURE 2.**
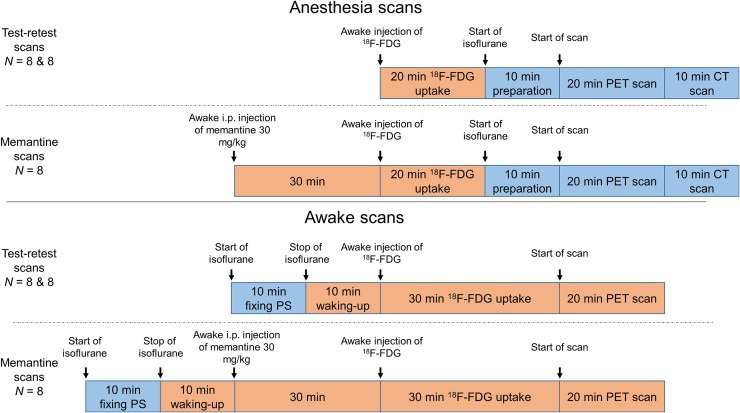
Scanning protocol for anesthetized and awake animals for test, retest, and memantine-challenge conditions. i.p. = intraperitoneal; PS = point sources; blue = anesthesia; orange = no anesthesia.

The animals were divided into 2 groups. One group of 8 mice (C57BL/6 [Charles River] 24.9±1.9 g, 18 wk old) was scanned under anesthesia, and another group of 8 mice (24.6±1.4 g, 18 wk old) was scanned while awake. This group division was retained for all 3 acquisitions (test, retest, and memantine-challenge scans) undergone by each animal. All animals were housed in a temperature-controlled room with a 12-h light–dark cycle (food and water available ad libitum). The night before each scan, all animals were kept fasting for at least 12 h with free access to water. Isoflurane gas anesthesia was used for the procedures (5% induction, 2% maintenance). The experiments followed the European Ethics Committee recommendations (decree 86/609/CEE) and were approved by the Animal Experimental Ethical Committee of the University of Antwerp, Antwerp, Belgium (ECD 2016-89). The protocol timeline for the anesthetized-mouse and awake-mouse scans is presented in Figure 2.

### Test–Retest Scans

For the test scans of the anesthetized group (*n* = 8, first row of Fig. 2), awake mice received an injection of 0.2 mL of ^18^F-FDG (18.5±0.66 MBq) in the tail vein. The mice were then placed in their home cages for an uptake period of 20 min. Afterward, the mice were anesthetized and placed on the scanner bed. The acquisition started at 30 min after tracer injection, with a duration of 20 min. Retest scans were performed 7 d after the test scans, following the same scanning protocol (^18^F-FDG, 19.0±0.65).

The test scans of the awake group (*n* = 8, third row of Fig. 2) followed the same protocol as for the anesthetized group, with 2 exceptions: the use of isoflurane anesthesia 20 min before tracer injection (19.0±0.44 MBq) for fixing the point sources (10-min duration), and no use of anesthesia to perform the PET acquisitions. Retest scans followed the same protocol (17.8±1.08 MBq) and were performed 7 d after the test scans.

### Memantine-Challenge Scans

Mice from the anesthetized group were scanned 3 d after the retest scans (second row of Fig. 2). The mice first received an intraperitoneal injection of memantine (30 mg/kg; Sigma Aldrich), after which they were returned to their cages for 30 min. These awake animals then received an ^18^F-FDG injection (18.6±0.50 MBq) through the tail vein. The mice were once again returned to their cages for an uptake period of 20 min. They were subsequently anesthetized with isoflurane and scanned for 20 min.

In a similar procedure, the mice in the awake group were scanned 3 d after the retest scans (fourth row of Fig. 2) following the same protocol as used for the anesthetized group and with the same 2 exceptions as indicated above (17.7±1.99 MBq).

Two mice from the anesthetized group (retest and memantine scans) and one from the awake group (memantine scan) were not properly injected with tracer or memantine. Additionally, one mouse removed a point source (awake-mouse retest scan). These animals were therefore excluded from the analysis.

### Brain Image Quantification

All image processing was performed in PMOD, version 3.6 (PMOD Technologies Ltd.).

First, the brain was cropped from the PET images and rigidly matched to a predefined ^18^F-FDG reference template in Waxholm space (18). The images were then spatially normalized through nonrigid registration. Predefined segmented brain regions in the reference template, delineated from an MR image, were used to calculate the mean regional ^18^F-FDG uptake (kBq/cm^3^) in the cortex, caudate putamen, thalamus, hippocampus, and cerebellum. SUVs for each brain region were calculated.

In addition, time–activity curves for each brain region were extracted from the dynamic reconstructions.

### Statistical Analysis

The SUV_mean_ and coefficient of variation for each brain region in the test, retest, and memantine-challenge conditions were calculated for the anesthetized and awake groups. Differences between conditions were assessed using 2-way ANOVA with Bonferroni adjustment.

Test–retest variability was calculated by comparing the regional brain uptake in the test and retest scans for each mouse. The intraclass correlation coefficient was calculated, and the Bland–Altman analysis performed, for each brain region.

A linear least-squares fit to the regional 20-min time–activity curves (30–50 min after injection of ^18^F-FDG) was performed. Slopes significantly different from 0 were determined using a *t* test. Statistical analysis was performed using GraphPad Prism, version 6.0 (GraphPad Software).

### Motion Analysis

For awake-mouse acquisitions, the average speed of the mouse was calculated based on the point (inside the head circumference) defined by the centroid of the point sources. The distanced traveled by the mouse was calculated for the total duration of the scan. Position histogram heat maps of the animal’s locations in the horizontal plane of the scanner FOV were calculated.

## RESULTS

### Brain Image Quantification

**FIGURE 3.**
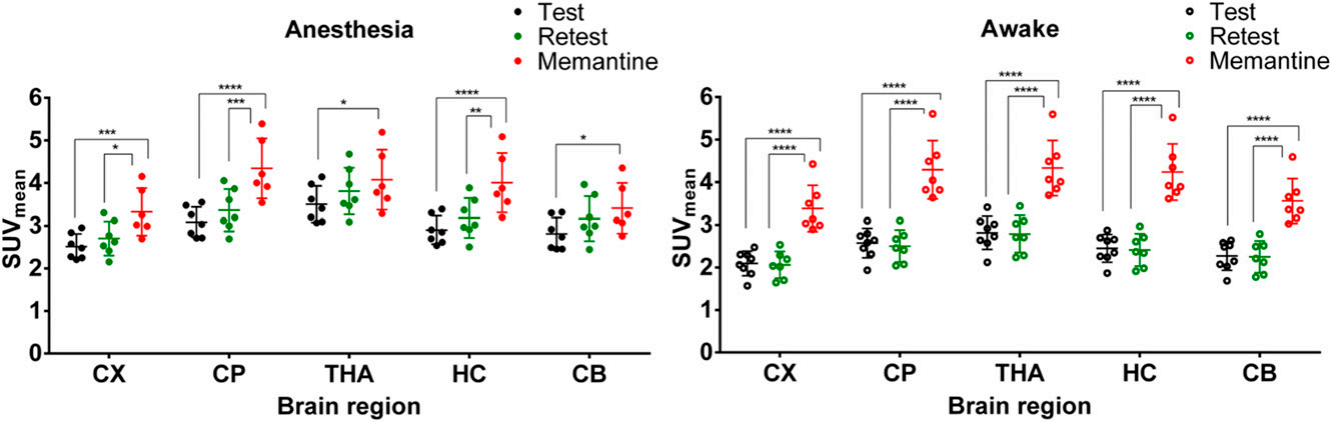
Scatterplot of SUV for different brain regions in test, retest, and memantine-challenge conditions for both anesthetized and awake groups. *P < 0.05. **P < 0.01. ***P < 0.001. ****P < 0.0001. CX = cortex; CP = caudate putamen; THA = thalamus; HC = hippocampus; CB = cerebellum.

Figure 3 shows the regional SUV for all conditions in anesthetized and awake animals. For the anesthetized animals, there was a significant difference (*P* < 0.05) between the memantine and the test and retest conditions in all tested regions, except for the thalamus and cerebellum in the retest condition. For the awake group, there was a highly significant difference (*P* < 0.0001) in all brain regions between the test–retest and the memantine-challenge conditions. In the thalamus, the SUV increased by 14.2% (*P* = 0.031) in the anesthetized group, whereas it increased by 51.5% (*P* < 0.0001) in the awake group. No significant differences were found between test and retest scans. However, there seemed to be a trend toward an increased uptake in the retest scan in the anesthetized group and a less pronounced trend toward a reduced uptake in the retest scan of the awake group (Supplemental Figs. 4 and 5; supplemental materials are available at http://jnm.snmjournals.org).

Within the anesthetized group, the coefficient of variation of the cortex was 11.8%, 14.8%, and 16.7% in the test, retest, and memantine scans, respectively. For the awake group, the coefficient of variation of the cortex was 13.8%, 15.3%, and 16% in the test, retest, and memantine scans, respectively. A similar trend could be observed for the other regions. The regional brain SUV_mean_ and coefficient of variation for all conditions in the anesthetized and awake groups are summarized in Table 1.

**TABLE 1:** Mean Regional SUV and Coefficient of Variation for Different Conditions in Anesthetized and Awake Animals

	SUV			Coefficient of variation (%)		
Brain region	Test	Retest	Memantine	Test	Retest	Memantine
Anesthetized						
Cortex	2.51	2.70	3.32	11.8	14.8	16.7
Caudate putamen	3.08	3.36	4.34	11.4	14.7	16.2
Thalamus	3.50	3.81	4.08	12.2	14.4	17.3
Hippocampus	2.90	3.18	4.01	11.4	14.7	17.4
Cerebellum	2.81	3.16	3.41	13.5	16.6	17.3
Awake						
Cortex	2.10	2.06	3.38	13.8	15.3	16.0
Caudate putamen	2.57	2.50	4.29	13.5	15.0	16.0
Thalamus	2.81	2.79	4.33	13.8	15.6	15.0
Hippocampus	2.45	2.41	4.24	13.3	15.8	15.6
Cerebellum	2.27	2.25	3.56	14.9	16.6	14.8

When the anesthetized and awake groups were compared, the SUV_mean_ in test and retest scans tended to be lower for awake animals. No significant differences between the anesthetized and awake mice (*P* > 0.999 for all brain regions) were found for the memantine challenge. A similar result could be observed for the time–activity curves for the anesthetized and awake groups, as displayed in Supplemental Figures 1 and 2 for the test and memantine conditions, respectively.

The test–retest variability statistics are presented in Table 2. The intraclass correlation coefficient in anesthetized-mouse scans was in the range of 0.491–0.629 for the different brain regions. In the awake-mouse scans, the intraclass correlation coefficient ranged from 0.424 to 0.555. The bias in the Bland–Altman analysis was largest in the anesthetized group for all brain regions, whereas the bias in the Bland–Altman SD was largest in the awake group.

**TABLE 2:** Intraclass Correlation Coefficient and Bland–Altman Bias and Bias SD for Test–Retest Comparison of Regional Brain SUV in Anesthetized and Awake Animals

Brain region	Intraclass correlation coefficient	Bias (%)	Bias SD
Anesthetized			
Cortex	0.629	−6.94	5.90
Caudate putamen	0.553	−8.25	5.94
Thalamus	0.580	−8.15	5.35
Hippocampus	0.505	−8.81	7.39
Cerebellum	0.491	−11.4	6.48
Awake			
Cortex	0.488	0.90	20.0
Caudate putamen	0.481	2.34	18.6
Thalamus	0.424	−0.04	19.2
Hippocampus	0.555	0.43	19.4
Cerebellum	0.444	−0.53	21.4

**FIGURE 4.**
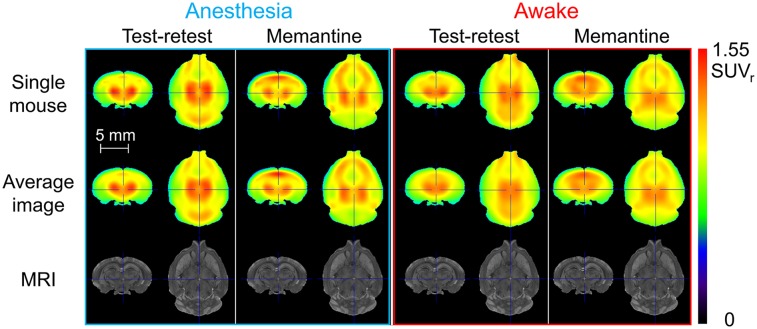
Anesthetized-mouse and awake-mouse images for a single mouse and averaged over all mice from each group. MRI template images are included as anatomic reference. Test–retest and memantine-challenge conditions are displayed for each group. Image values are normalized to whole-brain uptake for visualization purposes. SUV_r_ = SUV ratio.

The PET reconstructions after registration to the mouse brain template for a single mouse and the average of all reconstructions in anesthetized and awake mice for the test–retest and memantine-challenge conditions are displayed in Figure 4. The spatial resolution of the images was lower for the awake group than for the anesthetized group. On average, the awake-mouse images exhibited blurring comparable to the anesthetized-mouse images filtered with a gaussian filter with a σ of 0.6 mm. Both awake and anesthetized animals showed similar uptake patterns, as well as a memantine-induced change in the uptake pattern. The most striking memantine-induced changes included a reduced uptake in the cerebellum and an increased uptake in the hippocampus, relative to the other parts of the brain.

**FIGURE 5.**
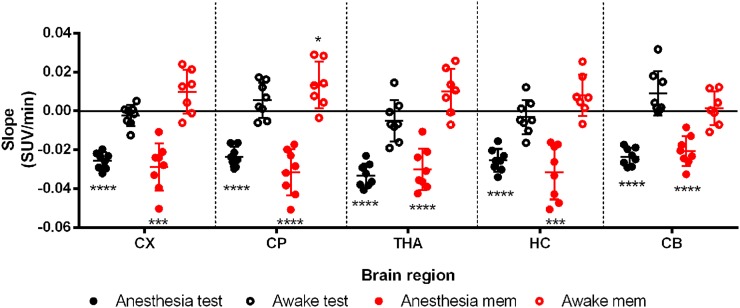
Slopes of linear least-squares fit to time–activity curves for anesthetized and awake groups in test and memantine-challenge conditions. *P < 0.05. ***P < 0.001. ****P < 0.0001. CX = cortex; CP = caudate putamen; THA = thalamus; HC = hippocampus; CB = cerebellum.

For the anesthetized animals, time–activity curve slopes decreased (*P* < 0.001) in all regions for all conditions (Fig. 5, Supplemental Figs. 1 and 2). During the 20-min scan, the SUV decreased by an average of 16% and 15% in the anesthesia and memantine-challenge conditions, respectively. For the awake animals, the slopes were not significantly different from zero, except for the caudate putamen, where there was an increase of 7% (*P* = 0.026) in the memantine-challenge condition.

### Motion Analysis of Awake Mice

**FIGURE 6.**
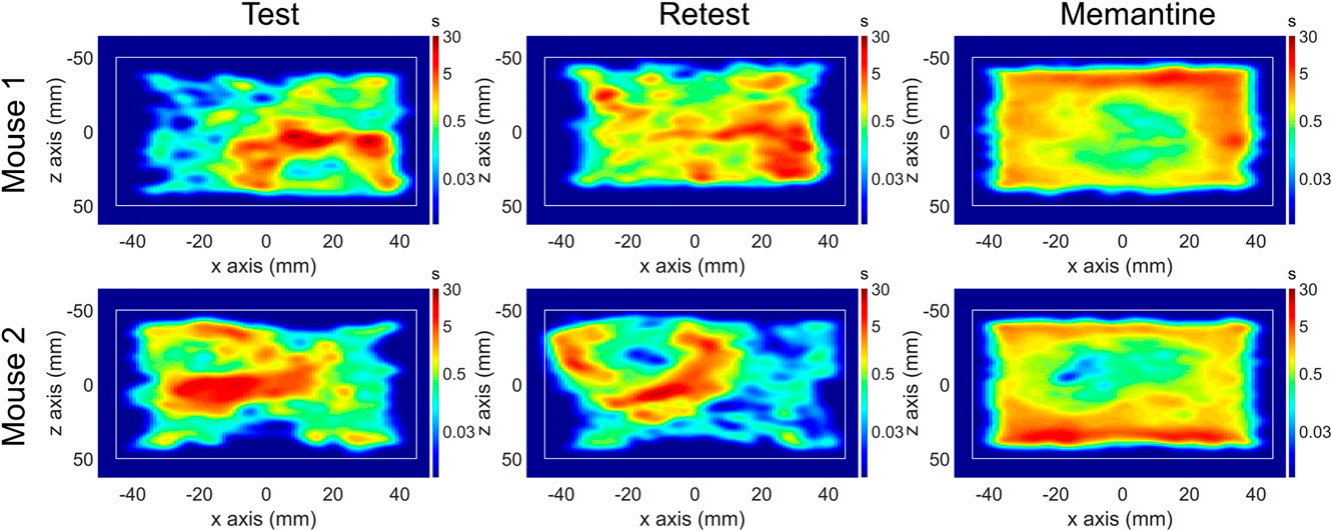
Position histogram (heat map) on horizontal plane for 2 mice during test, retest, and memantine-challenge acquisitions. Logarithmic time color scale is in seconds. Platform limits are represented as white rectangle.

Average head speed increased in the memantine-challenge condition (4.25±0.67 cm/s) relative to the test–retest condition (2.09±0.66 cm/s). There was a significant (*P* < 0.0001) increase in total distance traveled during the 20-min scan between the memantine (18.2±5.28 m) and test–retest (6.49±2.21 m) conditions. Figure 6 shows horizontal heat maps during the test, retest, and memantine-challenge scans for 2 representative animals. During test and retest scans, mice remained at the same location for longer periods, changing position sporadically. In contrast, during the memantine-challenge scans, mice moved constantly throughout the entire scan, with practically no resting periods. Supplemental Videos 1 and 2 show a mouse during a test scan, and Supplemental Videos 3 and 4 show a mouse during a memantine-challenge scan. The entire mouse ^18^F-FDG activity projection on the horizontal plane in short time frames (32 ms) is shown in Supplemental Videos 2 and 4.

## DISCUSSION

In this study, the PST method, which was previously developed for awake-rat brain PET scanning, was adapted for head motion tracking in awake mice. We adapted the algorithm developed for rat motion tracking to handle the faster movements of mice. In previous experiments, rat head-moving speed was around 0.5 cm/s (9), whereas mouse head-moving speed was around 2 cm/s in the test–retest experiments and 4 cm/s in the memantine-challenge experiments. Another modification compared with rat tracking was the use of a lightweight spacer to avoid spillover to the brain.

Similar to awake-rat imaging, some loss of spatial resolution could be observed in our awake-mouse images. Motion-tracking errors constitute one factor that causes deterioration in the spatial resolution of awake-animal images. For PST, these errors are caused primarily by slipping of the point sources on the skin of the mouse because of grooming behavior. Grooming might be reduced by familiarizing the mice to the awake-scan setup, especially with regard to the markers attached to the fur. A second factor that deteriorates the spatial resolution of awake images is the parallax effect. The position of the brain of an awake mouse is often located in off-center positions, which are closer to the edge of the FOV. In this case, the spatial resolution of the PET camera is lower than the centered position of an anesthetized animal.

Despite the loss of spatial resolution in awake-mouse images, the regional brain quantification was minimally affected. After the anesthesia images in the 3 conditions were filtered with a gaussian filter equivalent to the loss of spatial resolution in awake images (σ = 0.6 mm), no significant difference was found in regional brain quantification with respect to the original (unfiltered) image (Supplemental Fig. 3). In addition, the test–retest variability in these awake-mouse images was similar to that of scans performed using anesthesia, as quantified with the intraclass correlation coefficient and the Bland–Altman analysis. In a previous study, Casteels et al. (19) reported on the test–retest variability for ^18^F-FDG SUV brain regional quantification in anesthetized mice. The test–retest variability in the current study (e.g., in cortex and following a previously used metric (19): variability of 7.64% [anesthesia] and 14.15% [awake]) is lower than that reported in their study (27.74%).

In this study, we considered the irreversible tracer ^18^F-FDG to allow comparison between the awake-mouse and anesthetized-mouse PET acquisitions. The glucose analog ^18^F-FDG becomes trapped in the cell on phosphorylation by hexokinase (20). Given that the anesthetized-mouse scans had an awake uptake period similar to that of the awake-mouse scans, we expected the uptake in the two to be similar. Despite the anticipated similarities, anesthesia continued to have a large effect, as visible in the SUV quantification. First, the uptake in test–retest scans was lower for awake mice than for anesthetized mice. The brief administration of isoflurane 20 min before ^18^F-FDG injection to glue the point sources for awake-mouse acquisitions might have contributed to this effect (3,4). In future studies, a longer recovery period should be considered after fixing the point sources and before tracer injection. In the test–retest scans, the recovery period was only 10 min. A longer recovery period is necessary to allow brain function to return to normal. To this end, we extended the recovery period to 40 min for the memantine-challenge study. As a result, only a minimal difference between the awake and anesthetized SUVs (^18^F-FDG uptake in awake mice) was observed at the start of the scan, as could be seen from the regional time–activity curves (Supplemental Fig. 2). If longer recovery periods are required, the user should increase the activity of the point sources to account for the decay time.

In the retest scans, compared with the test scans, there was a trend toward an increased SUV in the anesthetized group and a decreased SUV in the awake group. If confirmed, these trends might be caused by anesthesia effects or differences in stress levels between repeated scanning. Indeed, in rodents it has been shown that ^18^F-FDG uptake quantified by SUV can be altered by repeated measurements (21). Although we allowed a 1-wk recovery period between the two scans, the repeated scanning might still have contributed to different trends.

We also observed a steady state (slope ≈ 0) in the time–activity curves during the 20-min scan in awake animals for both the test–retest and memantine conditions. This observation is similar to results reported by Mizuma et al. (4) 30 min after tracer injection. In contrast, ^18^F-FDG uptake in anesthetized mice decreased during the acquisition (∼15% during 20 min) for all conditions. This indicates that even after an awake uptake period, the changes in ^18^F-FDG consumption, induced by isoflurane, exert an effect on the ^18^F-FDG signal.

In both the anesthetized and the awake groups, we observed an increase in regional brain ^18^F-FDG uptake after memantine injection, in comparison to test–retest scans. On average, the difference between the test–retest and memantine challenges was 2.6 times larger for awake-mouse scans. This is also reflected in the significance levels and can be attributed to the effect of isoflurane. Our results reveal a trend toward increasing ^18^F-FDG uptake over time in the awake-mouse memantine condition for all regions, with the exception of the cerebellum.

In addition to the imaging, we assessed memantine-induced increased motor activity during PET acquisitions in the awake group. In the memantine challenge, the average mouse speed more than doubled relative to the test–retest condition. Moreover, the total distance traveled was about 3 times longer after memantine injection. The increase in mouse brain glucose uptake (11) has been reported elsewhere, as has the increase in motor activity (12) under memantine challenge. These results demonstrate the ability of the tracking algorithm to track the motion of animals in a wide range of speeds, as well as the ability of the awake-animal imaging to detect memantine-induced alterations in brain ^18^F-FDG uptake and changes in behavior.

We did not measure animal stress. A noninvasive stress quantification method is difficult to implement in freely moving mice and would most likely affect normal behavior. However, this study involved minimal sources of stress (e.g., handling of awake mice for tracer injections). Although the point sources were small enough to avoid distress in the animals, they seemed to cause mild stress in some mice (as evidenced by constant grooming behavior), whereas others were apparently unaffected (e.g., Supplemental Video 1). A training period might further reduce stress in the presence of the point sources.

Although ^18^F-FDG was used for validation purposes to compare the resulting images of awake versus anesthetized animals, awake-animal PET scans could be beneficial to other PET tracers. In addition to being a tool for studying the potential effects of the anesthesia brain kinetics of PET tracers, PET imaging of freely moving mice is particularly interesting for tracers whose pharmacokinetics might be correlated with animal behavior.

## CONCLUSION

In this study, test–retest and memantine-induced brain activation studies were performed on awake, freely moving mice. We generated reliable regional quantification for awake PET acquisitions. This reliability is reflected by slightly lower test–retest reproducibility in awake than anesthetized mice, along with very similar coefficients of variation for the two groups. Memantine-induced brain activation shows a clear increase in brain ^18^F-FDG uptake relative to unchallenged mice. In addition, the obtained behavioral information during the awake PET acquisitions showed increased motor activity during the memantine challenge. Our proposed PET setup makes it possible to study radiotracer brain uptake in awake animals, thereby ruling out the confounding factor of anesthesia and enabling the simultaneous measurement of animal behavior.

## DISCLOSURE

This work was supported by a Research Project (G0A8517N) and a Research Grant (1520217N) from the Research Foundation–Flanders (FWO). No other potential conflict of interest relevant to this article was reported.
