# Supplementary material for: Awake 18F-FDG PET Imaging of Memantine-Induced Brain Activation and Test–Retest in Freely Running Mice
Source: J Nucl Med. 2019 Jun;60(6):844–50. doi: 10.2967/jnumed.118.218669 (PMC6581220; doi:10.2967/jnumed.118.218669)
Supplement: Supplementary file 1 [file jnm218669SupplementalData.pdf]

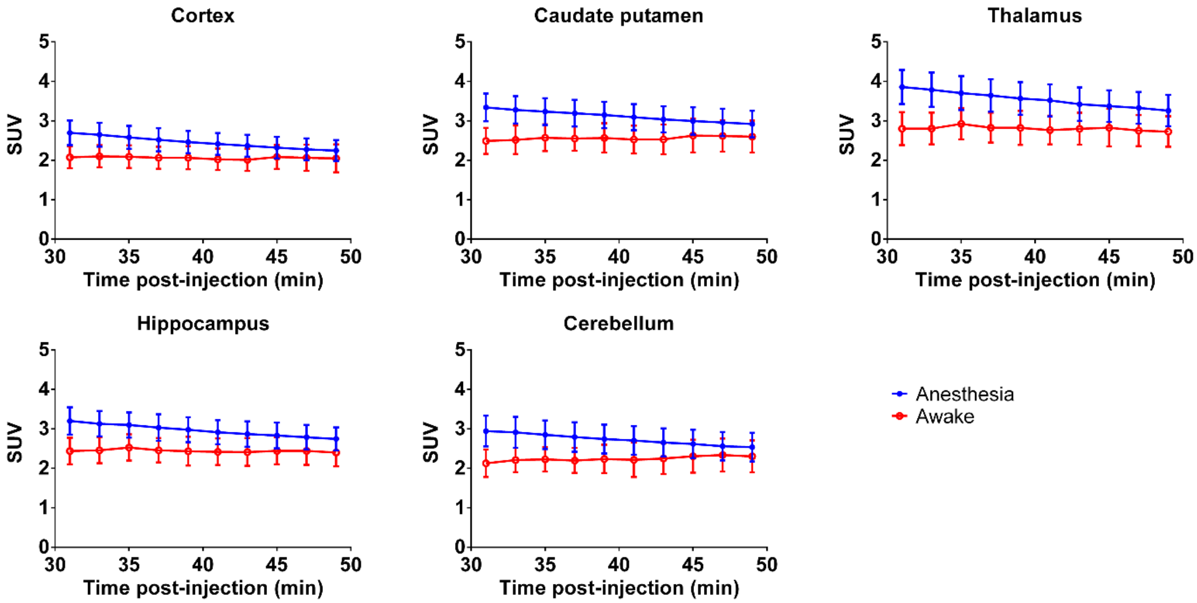

Supplemental Figure 1. Mean ± standard deviation time activity curves during the test scans for the different brain regions in the anesthesia (blue) and awake (red) group mice. Frame duration is 2 minutes.

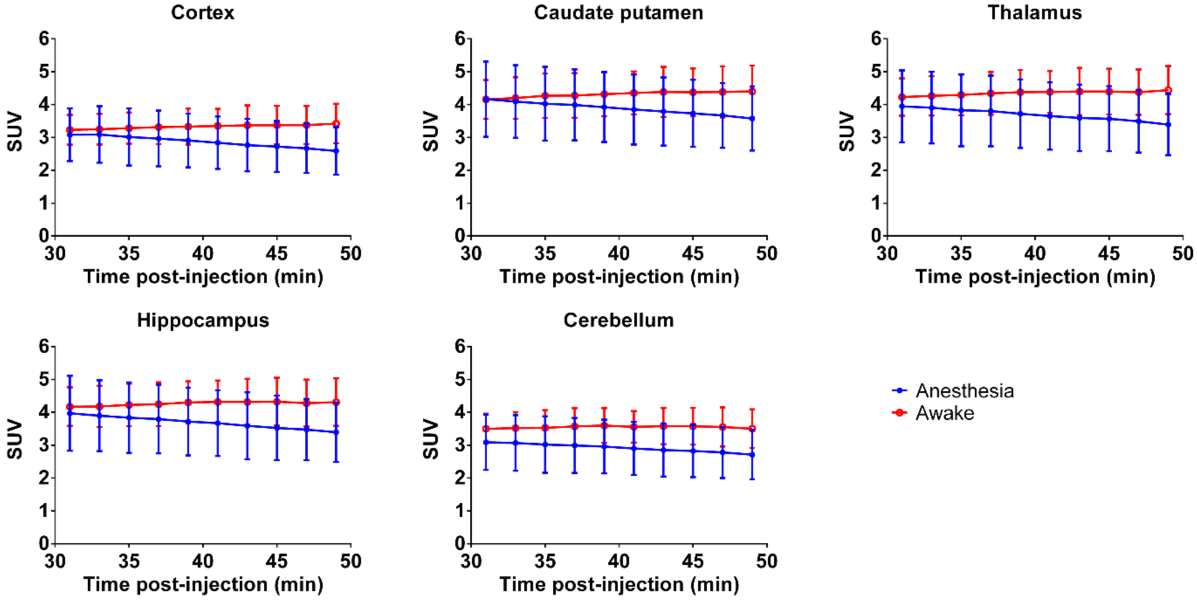

Supplemental Figure 2. Mean ± standard deviation time activity curves during the memantine challenge scans for the different brain regions in the anesthesia (blue) and awake (red) group mice. Frame duration is 2 minutes.

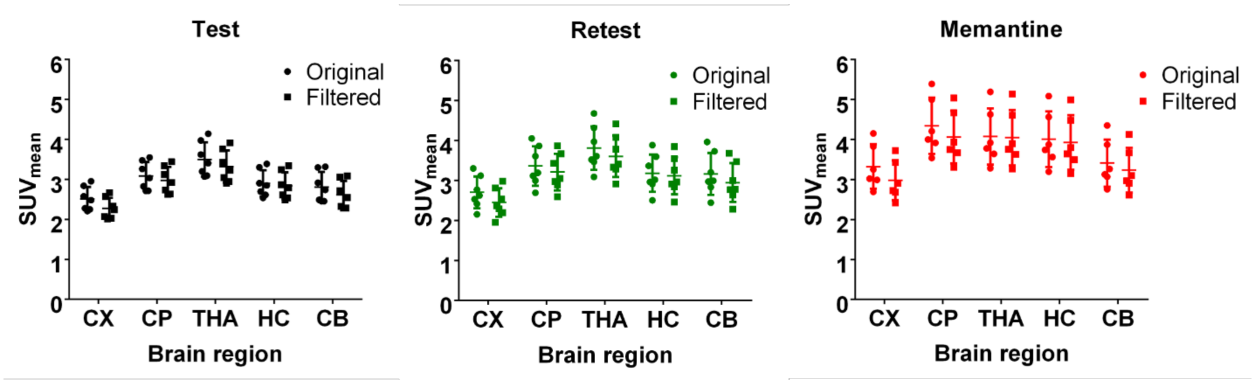

Supplemental Figure 3. Scatter plot of SUV for different brain regions for the test, retest and memantine condition for the anesthesia group original images (Original) and when smoothed with a Gaussian filter with σ = 0.6 mm (Filtered). No significant difference was found between regional brain quantification in original and filtered images in any case. CX: cortex, CP: caudate putamen, TH: thalamus, HC: hippocampus, CB: cerebellum.

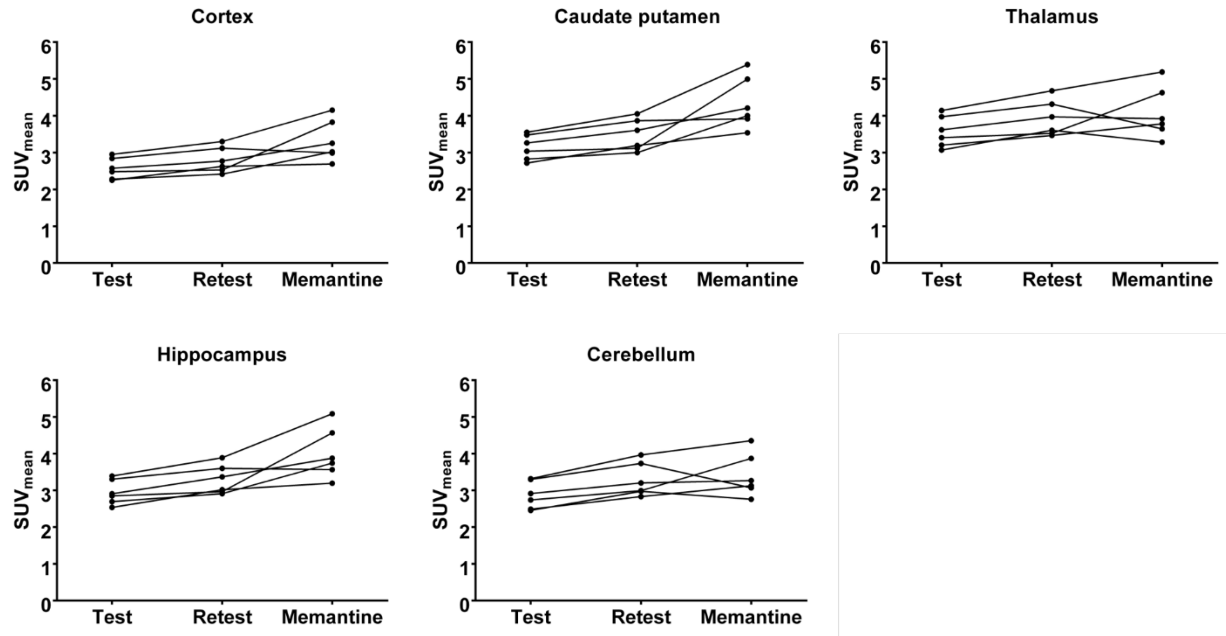

Supplemental Figure 4. Spaghetti plots of the SUV for different brain regions in the anesthesia group for the test, retest and memantine condition.

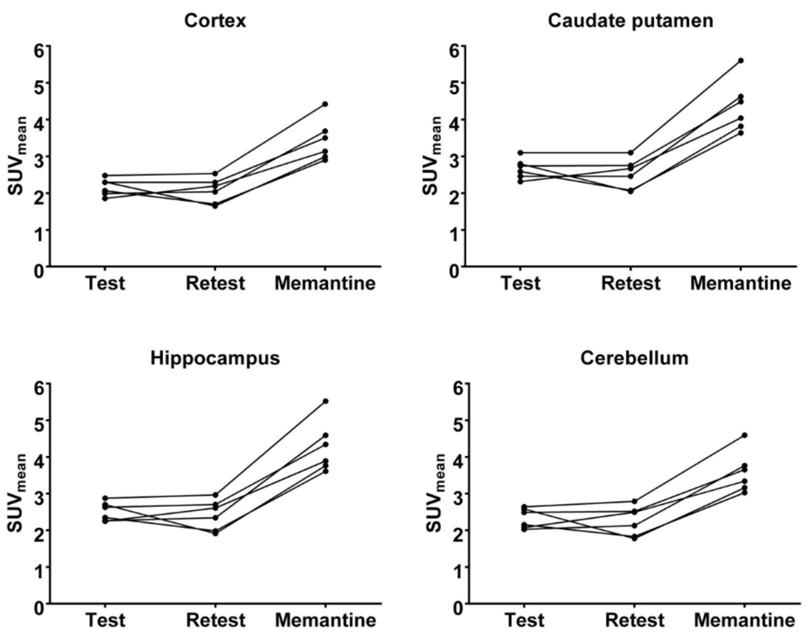

Supplemental Figure 5. Spaghetti plots of the SUV for different brain regions in the awake group for the test, retest and memantine condition.
